# Disease-associated H58Y mutation affects the nuclear dynamics of human DNA topoisomerase IIβ

**DOI:** 10.1038/s41598-022-24883-2

**Published:** 2022-11-30

**Authors:** Keiko Morotomi-Yano, Yukiko Hiromoto, Takumi Higaki, Ken-ichi Yano

**Affiliations:** 1 Institute of Industrial Nanomaterials, Kumamoto University, Kumamoto, Japan; 2 Faculty of Science, Kumamoto University, Kumamoto, Japan; 3 Faculty of Advanced Science and Technology, Kumamoto University, Kumamoto, Japan; 4 International Research Organization for Advanced Science and Technology, Kumamoto University, Kumamoto, Japan

**Keywords:** Mechanisms of disease, DNA-binding proteins, Nucleoproteins, Fluorescence imaging

## Abstract

DNA topoisomerase II (TOP2) is an enzyme that resolves DNA topological problems and plays critical roles in various nuclear processes. Recently, a heterozygous H58Y substitution in the ATPase domain of human TOP2B was identified from patients with autism spectrum disorder, but its biological significance remains unclear. In this study, we analyzed the nuclear dynamics of TOP2B with H58Y (TOP2B H58Y). Although wild-type TOP2B was highly mobile in the nucleus of a living cell, the nuclear mobility of TOP2B H58Y was markedly reduced, suggesting that the impact of H58Y manifests as low protein mobility. We found that TOP2B H58Y is insensitive to ICRF-187, a TOP2 inhibitor that halts TOP2 as a closed clamp on DNA. When the ATPase activity of TOP2B was compromised, the nuclear mobility of TOP2B H58Y was restored to wild-type levels, indicating the contribution of the ATPase activity to the low nuclear mobility. Analysis of genome-edited cells harboring TOP2B H58Y showed that TOP2B H58Y retains sensitivity to the TOP2 poison etoposide, implying that TOP2B H58Y can undergo at least a part of its catalytic reactions. Collectively, TOP2 H58Y represents a unique example of the relationship between a disease-associated mutation and perturbed protein dynamics.

## Introduction

DNA topoisomerase II (TOP2) is an ATP-dependent homodimeric enzyme that resolves DNA topological problems, such as torsional strain arising during transcription and DNA replication^1,2^. To relieve DNA topological problems, TOP2 utilizes a mechanism of DNA strand passage through a DNA double-strand break: TOP2 binds to two segments of DNA, introduces a double-strand break in one DNA segment, translocates the second DNA segment through a DNA break, and ligates the cleaved DNA^3,4^. A sequence of these reactions comprises a catalytic cycle, and various compounds have been developed to target specific steps of the cycle. TOP2 transiently forms a covalent complex with DNA ends during the catalytic cycle^4^. TOP2 poisons, such as etoposide, stabilize the covalent TOP2–DNA complex, which is readily converted into a DNA double-strand break in cells^4,5^. Thus, TOP2 poisons exhibit anti-proliferating activity and are used for cancer therapy. TOP2 catalytic inhibitors, such as ICRF-187, have a different mechanism of action: they trap TOP2 into a non-covalent complex with DNA, which is called a closed clamp^4,6,7^.

**Figure 1:**
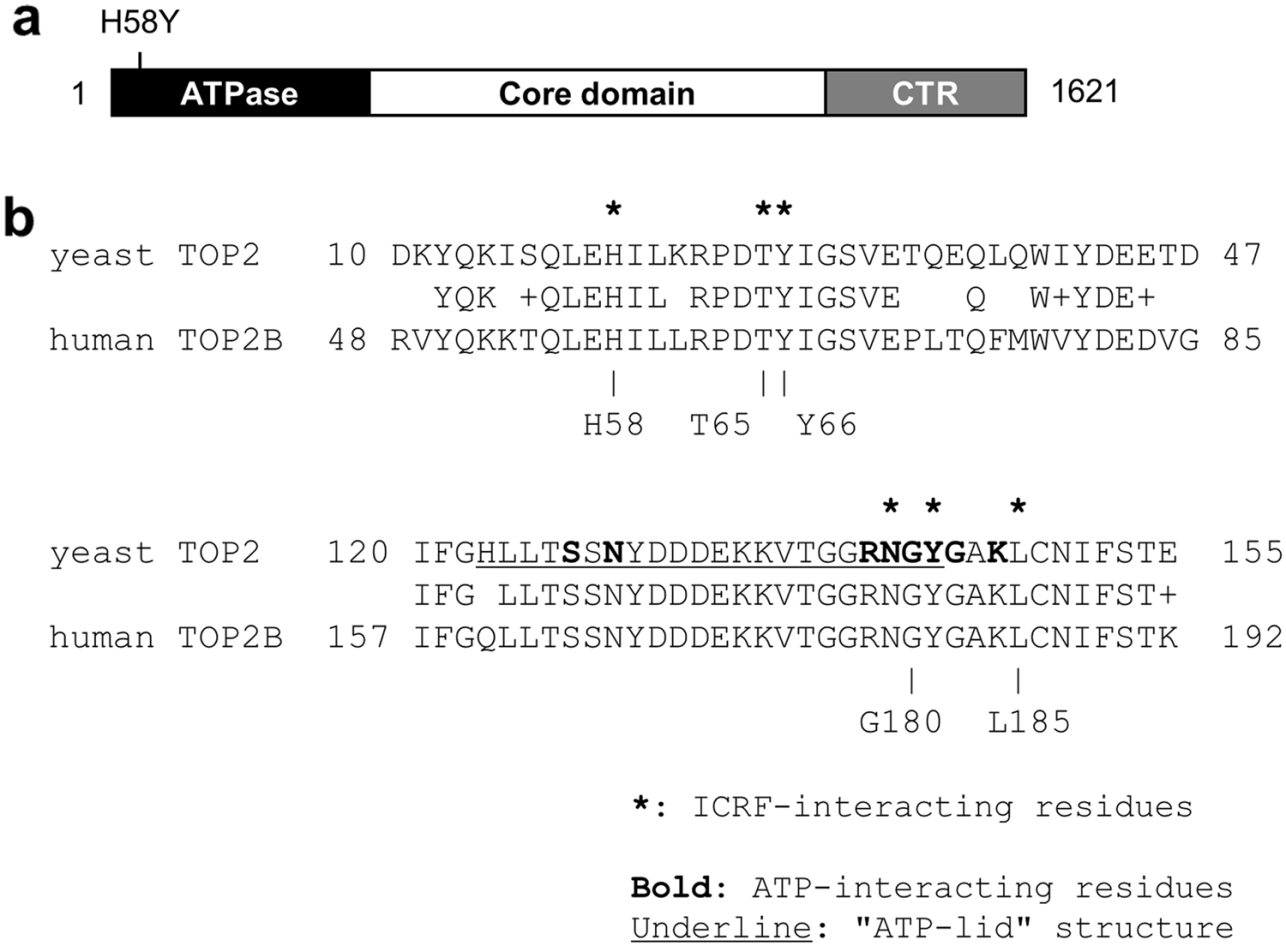
Structure of human TOP2B. (**a**) The domain structure of human TOP2B. Human TOP2B consists of 1621 amino acids that form an ATPase domain, a core catalytic domain, and a C-terminal region. (**b**) Sequence comparison of the N-terminal regions of yeast TOP2 (NCBI Reference Sequence: NP_014311.3) and human TOP2B (NCBI Reference Sequence: NP_001059.2). Asterisks on the yeast TOP2 sequence indicate amino acids that interact with ICRF-187. Bold letters in yeast TOP2 show amino acids interacting with ATP^48^. A region that forms an ATP-lid structure in yeast TOP2 is underlined^48^. Substitutions of T48I and Y49F in TOP2A of CHO cells were previously reported to confer ICRF resistance^48,49^. Their corresponding amino acids in human TOP2B are T65 and Y66, respectively. Substitutions of G180I and L185F confer ICRF resistance to human TOP2B^35,50^.

Eukaryotic TOP2 proteins have evolutionarily conserved domain structures: they consist of the N-terminal ATPase domain, the central catalytic core domain, and the C-terminal region^8–10^ (Fig. 1a). Humans and other vertebrates possess two TOP2, termed TOP2A and TOP2B^8,11^. The ATPase and core domains of human TOP2A and TOP2B share high sequence similarity, and the C-terminal regions are less conserved^9,10,12,13^. Although their catalytic properties in vitro are nearly indistinguishable^14,15^, TOP2A and TOP2B differentially participate in cellular functions^1,2^. TOP2A is expressed in proliferating cells with peak expression at G2/M of the cell cycle^16,17^. TOP2A plays critical roles in DNA replication and chromosome condensation/segregation and thus is essential for cell division^18,19^. TOP2B is expressed throughout the cell cycle and is present in both proliferating and non-dividing cells^17^. Although TOP2B is dispensable for cell proliferation, its physiological role is particularly evident in differentiated, non-dividing cells, such as neurons^20,21^. TOP2B is involved in the transcription of a distinct set of genes required for neural development^22–24^. Knockout of the TOP2B gene in mice causes various defects in neural development, leading to breathing impairment and consequent neonatal death immediately after birth^20^.

Although the complete loss of TOP2B functions leads to neonatal death in mice, individuals with partially dysfunctional TOP2B may remain viable. In general, perturbation of a particular activity of a multifunctional protein is more common than gross defects in its overall functions among human diseases caused by genetic disorders^25–27^. As for TOP2B, a number of germline missense mutations were identified as causative for human diseases. In the catalytic domain of TOP2B, the missense mutations, S483L, A485P, EE587E, and G633S, were identified in patients with B-cell deficiency^28–30^. Subsequent studies have demonstrated that these missense mutations in the catalytic domain lead to a significant decrease in the catalytic activity of TOP2B^28,29^.

Another pathogenic germline mutation was reported in the N-terminal ATPase domain of human TOP2B. H58Y was a heterozygous de novo mutation identified in patients with autism spectrum disorder and global developmental delay^31,32^. The patients carrying H58Y did not show any signs of B-cell deficiency, suggesting that the pathogenic impact of H58Y is different from those of the missense mutations in the central catalytic domains. H58Y is located in the N-terminal end of TOP2B and appears to have a relatively small impact on the TOP2B structure (Supplementary Fig. S1). We therefore imagined that H58Y exerts its deleterious effect on TOP2B functions other than the catalytic activity.

Previous studies have demonstrated that TOP2B is highly mobile in the nucleus of a living cell^33–37^. It is widely accepted that nuclear proteins, except for core histones, are highly mobile^38–41^. Dynamic nature is a common feature of most nuclear proteins, regardless of their biochemical properties or functional roles^39^. The vast majority of molecules of nuclear proteins rapidly move by stochastic diffusion. Only a small fraction of the molecules transiently reside in their sites of action, typically on the order of seconds^38–40,42^. For instance, more than 95% of molecules of transcription factors in the nucleus are estimated to be in diffusion or in transient nonspecific association with chromatin^40,42^. High mobility is considered to serve as a part of the mechanism for targeting proteins to appropriate sites and is also inferred to ensure the regulatory plasticity of various nuclear functions^40,41,43^. Importantly, alterations in the nuclear dynamics often reflect changes in the functional status of proteins^44–47^. In the case of TOP2B, its nuclear behavior readily alters in response to various cues, such as reduced ATP levels, DNA damage, and TOP2 inhibitors^35,37^, suggesting that the nuclear behavior of TOP2B reflects, at least in part, the status of TOP2B in living cells. In this viewpoint, we sought to examine whether the impact of H58Y may be reflected in the TOP2B dynamics. We found that H58Y confers a remarkable reduction in TOP2B mobility, representing an intriguing instance of the relationship between a disease-associated mutation and perturbed protein dynamics in living cells.

## Results

### Reduced nuclear mobility of TOP2B H58Y in living cells

**Figure 2:**
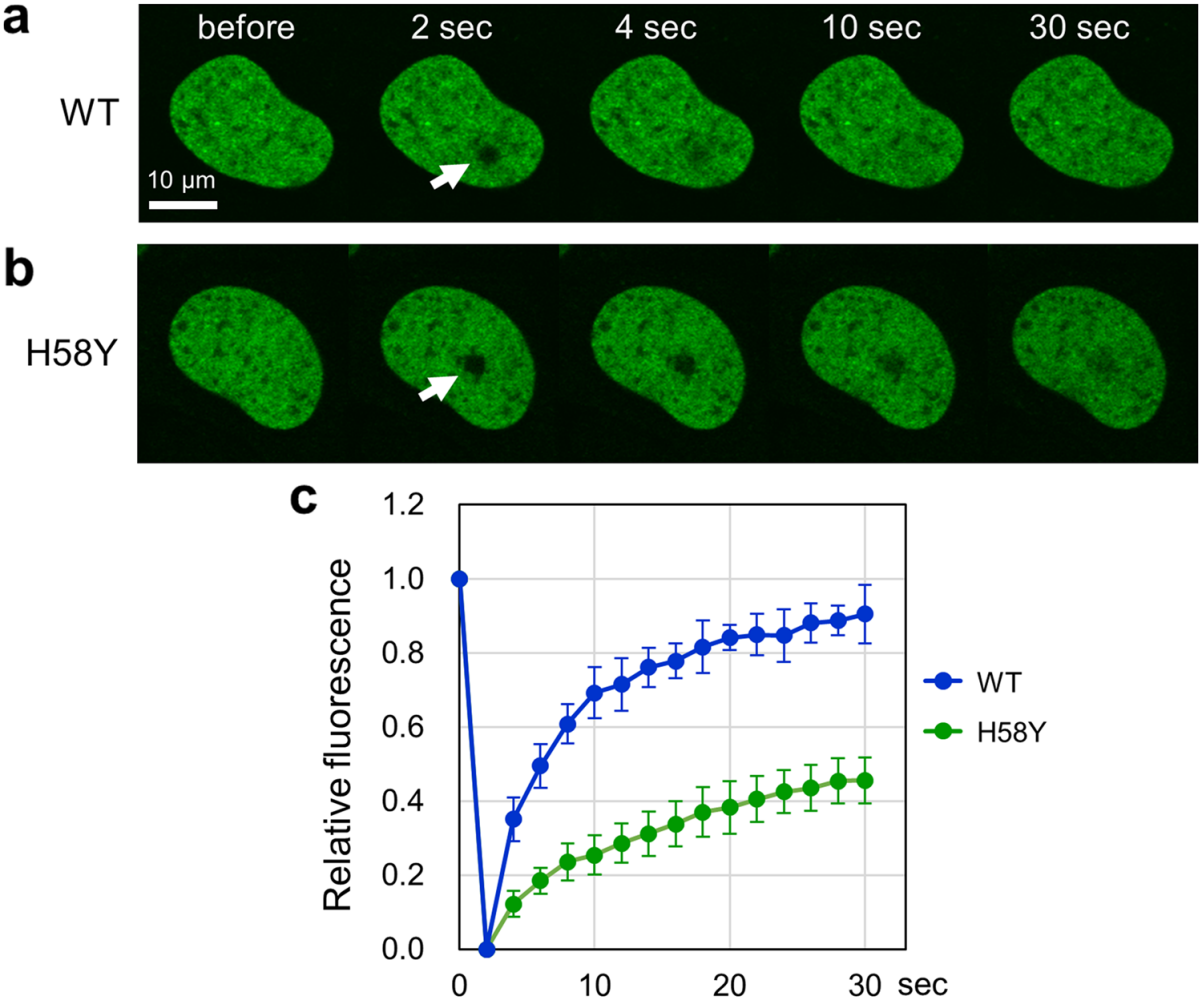
Reduced nuclear mobility of EGFP-TOP2B H58Y. (**a**) Representative images of FRAP analysis of EGFP-TOP2B WT. EGFP-TOP2B WT was transiently expressed in HeLa cells. A small area of the nucleus (shown with a white arrow) was photobleached, and fluorescence images were captured at the indicated time points. (**b**) Representative images of FRAP analysis of EGFP-TOP2B H58Y. FRAP analysis was performed on EGFP-TOP2B H58Y as described in (**a**). (**c**) Quantitative analysis of fluorescence recovery of EGFP-TOP2B WT and H58Y proteins. Fluorescence images were captured at 2 s intervals. Photobleaching was conducted at 2 s. Average values of relative fluorescence and SD were calculated (n = 10). The mobile fractions for WT and H58Y were estimated to be 0.87 ± 0.05 and 0.49 ± 0.05, respectively. The t1/2 of fluorescence recovery was calculated to be 3.73 ± 0.95 s (WT) and 8.81 ± 2.98 s (H58Y).

High mobility is a general feature of many nonhistone proteins^38–41^, and human TOP2B is also highly mobile in the nucleus^33–37^. We therefore investigated whether the H58Y substitution may affect the nuclear dynamics of TOP2B. Using HeLa cells transiently expressing EGFP-TOP2B, we performed FRAP analysis: green fluorescence in a small area in the nucleus was photobleached, and the fluorescence recovery was monitored over time. As shown in Fig. 2a, we observed the fast recovery of the fluorescence of wild-type TOP2B tagged with EGFP (referred to hereafter as EGFP-TOP2B WT) after photobleaching. This result confirmed that EGFP-TOP2B WT is highly mobile as reported previously^33–37^. Next, we carried out FRAP analysis on EGFP-TOP2B with the H58Y substitution (hereafter, EGFP-TOP2B H58Y). We observed that the fluorescence recovery was slow and partial as compared to EGFP-TOP2B WT (Fig. 2b). Quantification of fluorescence highlighted the marked difference between EGFP-TOP2B WT and H58Y (Fig. 2c). When compared at 30 s after photobleaching, the recovery of EGFP-TOP2B WT fluorescence reached approximately 90%, but that of EGFP-TOP2B H58Y was estimated to be less than 50% (Fig. 2c). These observations demonstrate that H58Y impacts the nuclear mobility of TOP2B in living cells.

### Insensitivity of TOP2B H58Y to ICRF-187

**Figure 3:**
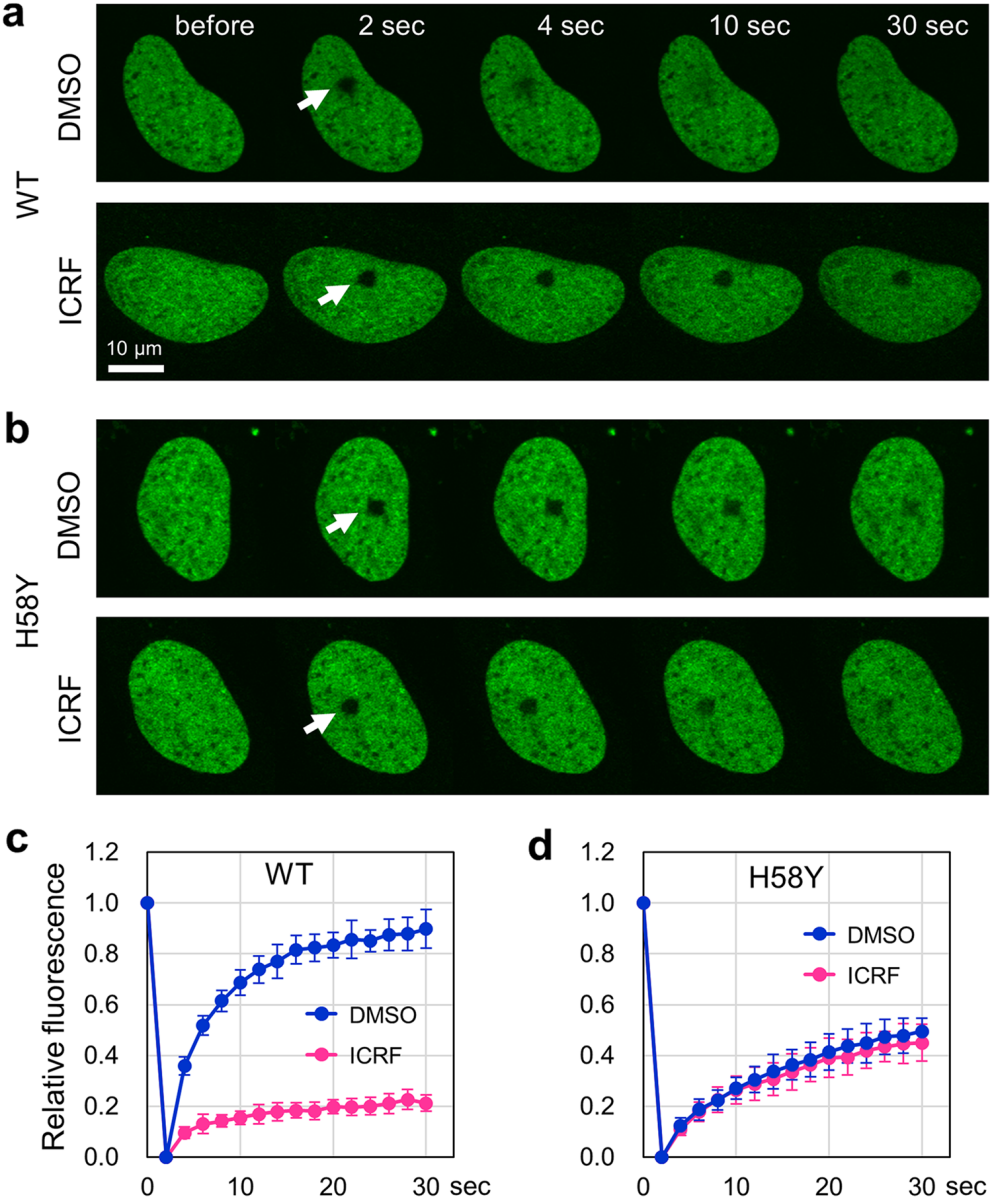
Insensitivity of EGFP-TOP2B H58Y to ICRF-187. (**a**) Representative images of FRAP analysis of EGFP-TOP2B WT in the presence of ICRF-187. Cells were treated with 20 µM ICRF-187 or DMSO (vehicle) for 1 h and subsequently subjected to FRAP analysis. White arrows indicate the area of photobleaching. (**b**) Representative images of FRAP analysis of EGFP-TOP2B H58Y in the presence of ICRF-187. Experiments with ICRF-187 were performed on EGFP-TOP2B H58Y as described in (**a**). (**c**) Quantitative analysis of fluorescence recovery of EGFP-TOP2B WT in the presence and absence of 20 µM ICRF-187 (n = 10; bars, SD). The mobile fractions were estimated to be 0.86 ± 0.06 (DMSO) and 0.20 ± 0.03 (ICRF). The t1/2 of fluorescence recovery was calculated to be 3.40 ± 0.34 s (DMSO) and 4.11 ± 1.75 s (ICRF). (**d**) Quantitative analysis of fluorescence recovery of EGFP-TOP2B H58Y in the presence and absence of 20 µM ICRF-187 (n = 10; bars, SD). The mobile fractions were estimated to be 0.55 ± 0.08 (DMSO) and 0.48 ± 0.06 (ICRF). The t1/2 of fluorescence recovery was calculated to be 9.54 ± 3.56 s (DMSO) and 8.21 ± 1.98 s (ICRF).

We attempted to further characterize the property of TOP2B H58Y in living cells. ICRF-187 is a catalytic inhibitor of TOP2 and traps TOP2 on a DNA strand as a closed clamp^4,7^. Previous studies have shown that treatment with ICRF-187 significantly reduces the nuclear mobility of TOP2B^35^. The N-terminal region of TOP2 is conserved between yeast TOP2 and human TOP2B, and H58 of human TOP2B corresponds to yeast TOP2 H20, which interacts with ICRF-187^48^ (Fig. 1b). We then inquired whether the H58Y substitution affects the sensitivity of TOP2B to ICRF-187 in living cells. As shown in Fig. 3a, treatment with ICRF-187 significantly suppressed the fluorescence recovery of EGFP-TOP2B WT in the bleached region. At 30 s after photobleaching, the fluorescence recovery of EGFP-TOP2B WT was approximately 20% (Fig. 3c). In contrast, treatment with ICRF-187 did not affect the fluorescence recovery of EGFP-TOP2B H58Y (Fig. 3b). Quantified values of FRAP analysis of EGFP-TOP2B H58Y were indistinguishable between ICRF-treated and untreated cells (Fig. 3d). These observations indicate that the H58Y substitution confers insensitivity of TOP2B to ICRF-187.

**Figure 4:**
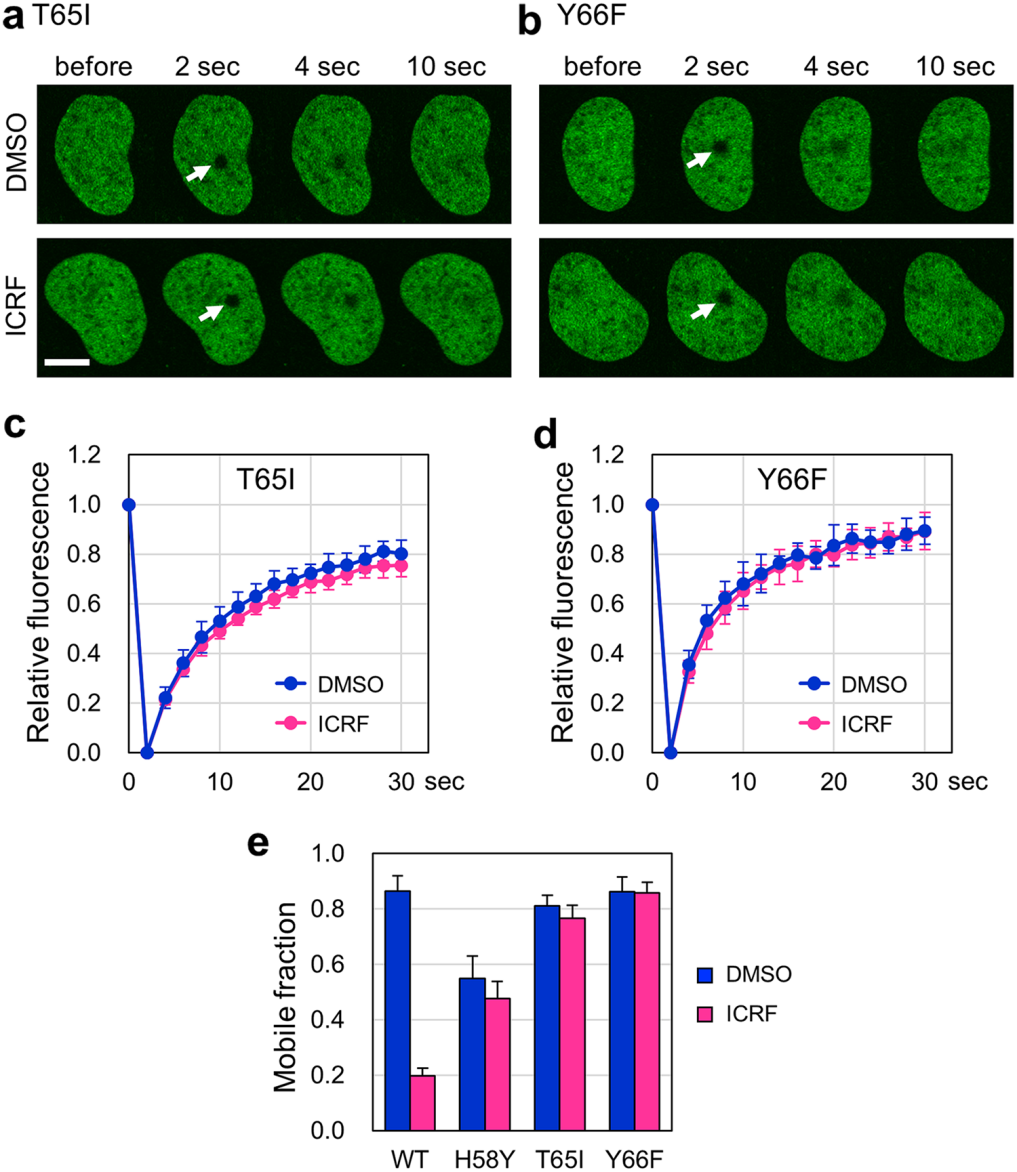
High nuclear mobility of ICRF-resistant T65I and Y66F proteins. (**a,b**) Representative images of FRAP analysis of EGFP-TOP2B T65I and Y66F in the presence of ICRF-187. Experiments were performed as described in Fig. 3a. (**c**) Quantitative analysis of fluorescence recovery of EGFP-TOP2B T65I in the presence and absence of 20 µM ICRF-187 (n = 10; bars, SD). The mobile fractions were estimated to be 0.81 ± 0.04 (DMSO) and 0.77 ± 0.05 (ICRF). The t1/2 of fluorescence recovery was calculated to be 5.36 ± 0.98 s (DMSO) and 5.66 ± 1.01 s (ICRF). (**d**) Quantitative analysis of fluorescence recovery of EGFP-TOP2B Y66F in the presence and absence of 20 µM ICRF-187 (n = 10; bars, SD). The mobile fractions were estimated to be 0.86 ± 0.05 (DMSO) and 0.86 ± 0.04 (ICRF). The t1/2 of fluorescence recovery was calculated to be 3.58 ± 0.87 s (DMSO) and 3.91 ± 0.60 s (ICRF). (**e**) Comparison of the mobile fractions. The graph represents the mobile fractions shown in Fig. 3c (WT), Fig. 3d (H58Y), (**c**) (T65I), and (**d**) (Y66F) (n = 10; bars, SD). Statistical values between DMSO and ICRF treatments are as follows; WT: p = 1.41E−14, H58Y: p = 0.043, T65I: p = 0.031, Y66F: p = 0.822.

We next asked whether the low nuclear mobility is a common feature of ICRF-insensitive TOP2B mutants. A previous study showed that TOP2A harboring either T48I or Y49F in CHO cells exhibits ICRF resistance^48,49^. T48 and Y49 of TOP2A of CHO cells correspond to T65 and Y66 of human TOP2B, respectively (Fig. 1b). We transiently expressed either EGFP-TOP2B T65I (Fig. 4a,c) or Y66F (Fig. 4b,d) and performed FRAP analysis with or without ICRF-187 treatment. As expected, these mutant TOP2B proteins were insensitive to ICRF-187, because the fluorescence recoveries of these proteins were largely unaffected by ICRF-187 (Fig. 4c–e). These mutants were highly mobile, and their nuclear mobilities were comparable to that of EGFP-TOP2B WT (Fig. 4e). These observations demonstrate that the marked reduction in the nuclear mobility is a characteristic feature of TOP2B H58Y and is not shared with other ICRF-insensitive mutants.

### Restoration of TOP2B H58Y mobility by G180I

**Figure 5:**
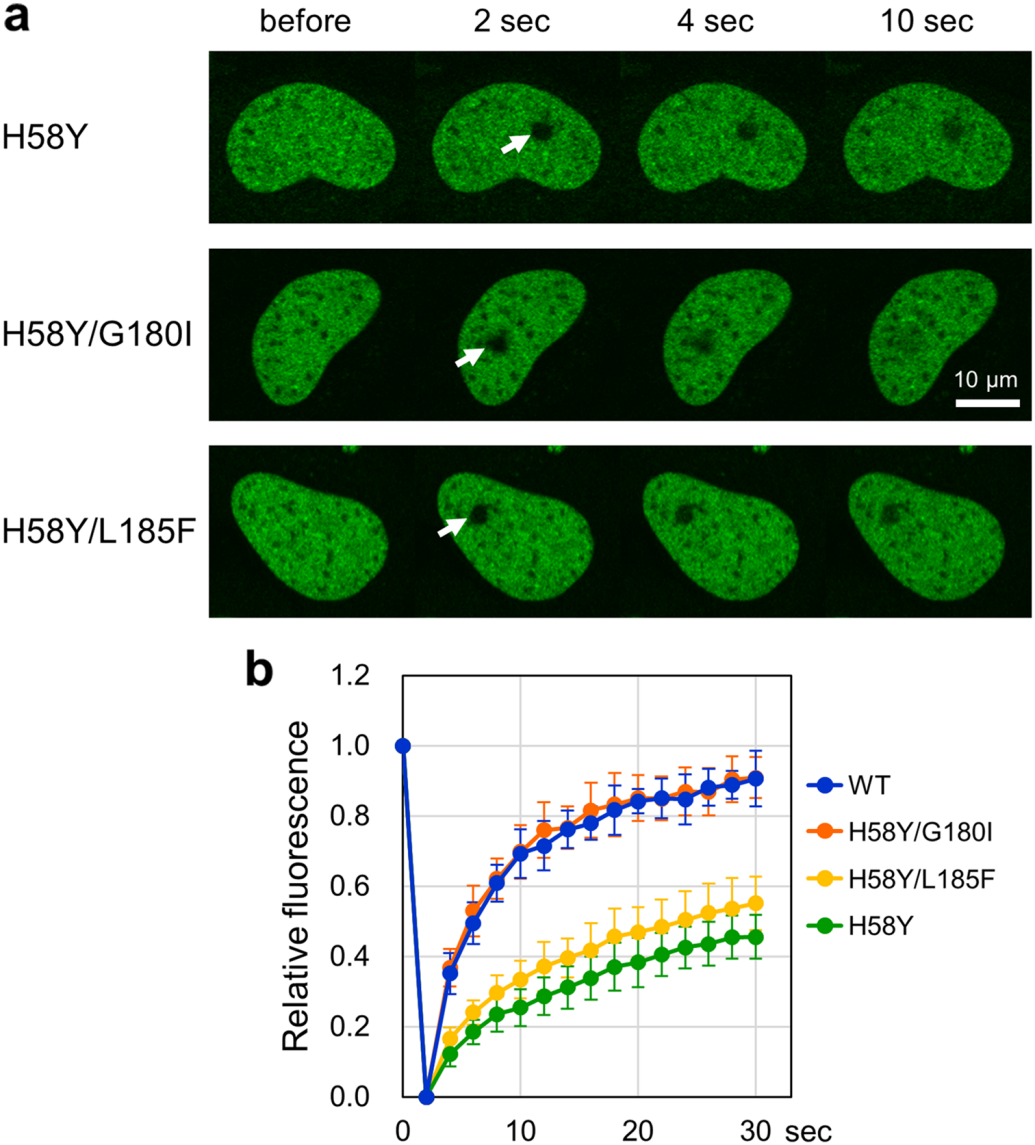
G180I restores the nuclear mobility of TOP2B H58Y. (**a**) Representative images of FRAP analysis of EGFP-TOP2B H58Y, H58Y/G180I, and H58Y/L185F. (**b**) Quantitative analysis of fluorescence recovery of EGFP-TOP2B WT, H58Y, H58Y/G180I, and H58Y/L185F (n = 10; bars, SD). The graphs of WT and H58Y are same as those in Fig. 2C. The mobile fractions were estimated as follows; WT: 0.87 ± 0.05, H58Y: 0.49 ± 0.05, H58Y/G180I: 0.87 ± 0.05, H58Y/L185F: 0.54 ± 0.08. The t1/2 of fluorescence recovery was calculated as follows; WT: 3.73 ± 0.95 s, H58Y: 8.81 ± 2.98 s, H58Y/G180I: 3.43 ± 0.69 s, H58Y/L185F: 6.60 ± 0.88 s.

TOP2B has an ATPase domain, and two amino acid substitutions in this domain, G180I and L185F, are known to confer ICRF insensitivity on TOP2B^35,50^. G180 is located in the ATP-lid structure (Fig. 1b), and the G180I substitution abolishes ATP-binding of TOP2B and consequently prevents the formation of the closed clump structure of ICRF-bound TOP2B on DNA strands^35,50,51^. TOP2B with L185F is ICRF-insensitive and partially retains the catalytic activity^35,50,52^. First, we used EGFP-TOP2B with both H58Y and G180I (EGFP-TOP2B H58Y/G180I) for FRAP analysis. We found that the fluorescence recovery of EGFP-TOP2B H58Y/G180I was similar to that of EGFP-TOP2B WT, demonstrating that G180I cancels the effect of H58Y on the nuclear mobility of TOP2B (Fig. 5a). We repeated FRAP analysis using EGFP-TOP2B with both H58Y and L185F and observed that L185F did not cancel the effect of H58Y on the mobility (Fig. 5a). Quantification of fluorescence recovery confirmed that G180I restores the nuclear mobility of TOP2B H58Y (Fig. 5b). These observations imply that the ATPase activity of TOP2B contributes to the reduced nuclear mobility of EGFP-TOP2B H58Y.

### Drug sensitivity of cells harboring heterozygous H58Y

**Figure 6:**
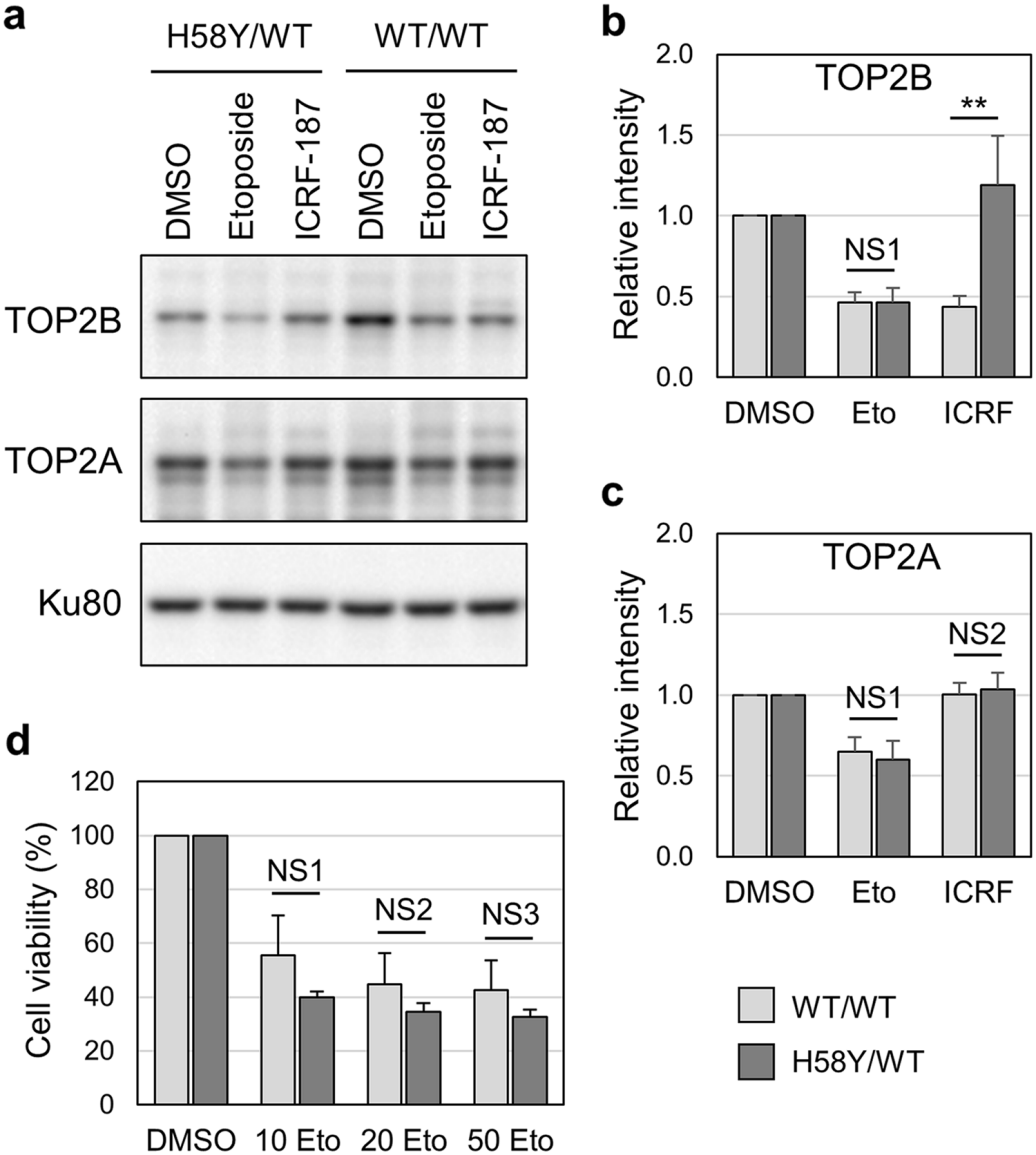
Effects of ICRF-187 and etoposide on TOP2B in H58Y/WT and WT/WT cells. (**a**) Western blot analysis of H58Y/WT and WT/WT cells. Cells were treated with either DMSO, 100 µM etoposide, or 20 µM ICRF-187 for 2 h. TOP2B and TOP2A were analyzed by Western blotting. Ku80 was shown as a loading control. Original uncropped images are shown in Supplementary Fig. S4. (**b**) Quantification of Western blot signals of TOP2B. Experiments were repeated 3 times as described in (**a**). Original uncropped images are shown in Supplementary Fig. S4. Values of DMSO-treated samples were arbitrarily set 1, and average values of relative intensities of western blot bands were calculated (n = 3; bar, SD). Statistical values are as follows; **: statistically significant; p = 0.0457. *NS* not significant. NS1; p = 0.976. (**c**) Quantification of Western blot signals of TOP2A. Experiments were repeated 3 times as described in (**a**). Original uncropped images are shown in Supplementary Fig. S4. Values of DMSO-treated samples were arbitrarily set 1, and average values of relative intensities of western blot bands were calculated (n = 3; bar, SD). Statistical values are as follows; NS; Not significant. NS1; p = 0.591, NS2; p = 0.664. (**d**) Effects of etoposide on the viabilities of H58Y/WT and WT/WT cells. Cells were treated with either DMSO, 10 µM, 20 µM, or 50 µM etoposide for 2 h. After washing with fresh media, cells were cultured for 3 days. Values of DMSO-treated samples were arbitrarily set to 100%. Average values with SD were calculated from 5 independent experiments. Statistical values are as follows; *NS* not significant. NS1; p = 0.113, NS2; p = 0.116, NS3; p = 0.124.

The H58Y substitution was originally identified as a heterozygous germline mutation in patients with autism spectrum disorder and global developmental delay^31,32^. We generated a cell line that harbors heterozygous H58Y in TOP2B by CRISPR/Cas9-mediated genome editing (Supplementary Fig. S2). Using this heterozygous H58Y cell line (H58Y/WT) and its parental HCT-116 (WT/WT), we performed immunofluorescence for TOP2B and observed that TOP2B was localized in the nucleus in both cell types (Supplementary Fig. S3). We next carried out Western blotting of the H58Y/WT and WT/WT cells to analyze the effects of etoposide on TOP2B (Fig. 6a,b) and TOP2A (Fig. 6a,c). If the covalent TOP2-DNA complex was formed by etoposide treatment, the Western blot signal should be reduced as reported previously^50^. We observed that the Western blot signal of TOP2B was decreased by etoposide treatment in both H58Y/WT and WT/WT cells (Fig. 6a). Quantified values of the Western blot signals of TOP2B were statistically indistinguishable between the H58Y/WT and WT/WT cells (Fig. 6b), suggesting that the etoposide sensitivity of TOP2B in the H58Y/WT cells was comparable to that in WT/WT cells. Next, we analyzed the effect of ICRF-187 on TOP2B by Western blotting. Previous studies have reported that ICRF treatment enhances the degradation of TOP2B, but not TOP2A^7,53^. Consistently, we observed a decrease in TOP2B, but not TOP2A, in the ICRF-treated WT/WT cells (Fig. 6b,c). In the H58Y/WT cells, ICRF-187 treatment did not cause a decrease of TOP2B in Western blotting, supporting the ICRF insensitivity of TOP2B in the H58Y/WT.

Lastly, we analyzed the effect of etoposide on the viabilities of WT/WT and H58Y/WT cells. As shown in Fig. 6d, there was no statistically significant difference in etoposide sensitivity between the WT/WT and H58Y/WT cells, although we observed a tendency for the H58Y/WT cells to exhibit slightly lower viability after etoposide treatment. Together with the results of immunofluorescence and Western blotting, our observations demonstrate that TOP2B in the H58Y/WT cells is insensitive to ICRF-187 but sensitive to etoposide.

## Discussion

In this study, we characterized the nuclear dynamics of TOP2B H58Y in comparison with TOP2B WT and other TOP2B mutants. TOP2B WT was highly mobile in the nucleus, which is consistent with the prevailing view of the high mobility of nonhistone proteins. In contrast, the nuclear mobility of TOP2B H58Y was remarkably low (Fig. 2). We also found that TOP2B H58Y was insensitive to ICRF-187 (Fig. 3). The low nuclear mobility was not shared with other ICRF-resistant mutants and thus regarded as a characteristic feature of H58Y (Fig. 4). G180I is known to abolish the TOP2B ATPase activity, and TOP2B with both H58Y and G180I behaved indistinguishably from TOP2B WT (Fig. 5), suggesting that the ATPase activity contributes to the low nuclear mobility of TOP2B H58Y. Taken together, these results revealed the prominent features of TOP2B H58Y from the viewpoint of nuclear dynamics in living cells.

H58Y was originally identified as a de novo germline mutation in patients with autism spectrum disorder and global developmental delay^31,32^. Although the low nuclear mobility is suggestive of a compromised state of TOP2B H58Y in living cells, further investigation is required for a more detailed understanding of the biological relevance of H58Y to pathogenesis. The first point to be elucidated in future research is the mechanism underlying the reduced mobility of TOP2B H58Y. We assume that TOP2B H58Y may tend to pause in the TOP2 catalytic cycle under some circumstances in living cells. As shown in Fig. 6a,b, etoposide treatment weakened the bands for TOP2B H58Y in the Western blotting. This means that TOP2B H58Y retains the catalytic activity, at least in part. More specifically, we speculate that TOP2B H58Y should be capable of going through its catalytic cycle from the initial DNA binding step to at least the step of religation of cleaved DNA ends, where etoposide acts^4^. The next step after the DNA religation is the reopening of the TOP2B closed clamp, which allows the release of TOP2B from DNA^4^. If TOP2B H58Y tends to be stalled at the reopening step of the catalytic cycle, this could lead to the low nuclear mobility of TOP2B H58Y. Of note, ICRF-187 inhibits the reopening step in an ATP-dependent manner^6,48^. ICRF-187 acts on an ATP-bound form of TOP2B. In the absence of ATP, ICRF-187 does not exert its inhibitory effect^6^. ATP-binding is also critical for the nuclear mobility of TOP2B H58Y, because the G180I substitution, which abolishes ATP-binding of TOP2B, restores the nuclear mobility of TOP2B H58Y (Fig. 5). Considering the similarity in ATP requirement, we speculate that the H58Y substitution may mimic the effect of ICRF-187. To test this idea, the impact of H58Y on the catalytic cycle of TOP2B should be evaluated using a purified protein in future research.

The second point to be investigated in the future is the molecular mechanism by which the H58Y substitution leads to the pathogenesis of autism. Previous studies demonstrated that TOP2B knockout results in multiple neurological defects^20,54^, indicating the critical role of TOP2B in neural development. TOP2B modulates the expression of a specific subset of neuronal genes^22–24^. The neurological defects in TOP2B knockout mice are currently ascribed to the perturbation of TOP2B-mediated gene expression^55^. Genome-wide analysis of TOP2B binding sites showed that TOP2B preferentially associates with the promoter regions of actively transcribed neuronal genes^22,56^, indicating the role of TOP2B in transcriptional initiation. In addition, a previous study demonstrated the involvement of TOP2B and TOP1 in transcription elongation of long genes in cultured cortical neurons^57^. Intriguingly, some of these TOP2B-controlled long genes are linked to autism^57^. Given the role of TOP2B in neuronal gene expression, the H58Y substitution may perturb the gene expression patterns during neural development, which may eventually lead to autism. Genome-wide analyses of gene expression and TOP2B binding sites in WT and H58Y-harboring neuronal cells will provide insights into the clinical relevance of H58Y.

Missense mutations are common genetic alterations found in human hereditary disorders, and gross functional defects caused by missense mutations are relatively rare in human diseases^25–27^. Instead, many missense mutations are detrimental to a limited extent and impair a specific protein function, while all other functional properties remain unaffected^25–27^. In the case of TOP2B, the gene knockout in mice results in neonatal death^20^, and therefore, disease-associated TOP2B mutations should restrain a part of the TOP2B functionality. TOP2B H58Y appears to be an intriguing example where a detrimental effect of a disease-associated missense mutation manifests as low protein mobility in living cells. Further research will advance our understanding of the molecular details of the impact of H58Y on TOP2B functions and the relevance to pathogenesis.

## Materials and methods

### Reagents and antibodies

ICRF-187 was obtained from Cayman Chemical (USA). Etoposide was purchased from FUJIFILM Wako Pure Chemicals (Japan). Following antibodies were used in this study: mouse anti-TOP2B antibody (#611492, BD Biosciences, USA), rabbit anti-TOP2A antibody (12286S, Cell Signaling Technology, USA), anti-mouse HRP-linked IgG (7076S, Cell Signaling Technology), anti-rabbit HRP-linked IgG (7074S, Cell Signaling Technology). Mouse anti-Ku80 antibody was a kind gift from Prof. David Chen (University of Texas Southwestern Medical Center at Dallas).

### Plasmids

The expression plasmid for EGFP-tagged human TOP2B (EGFP-TOP2B) was described previously^35^. Site-directed mutagenesis of the EGFP-TOP2B expression plasmid was performed using specific oligonucleotides to yield H58Y, T65I, Y66F, G180I, and L185F substitutions. Sequence information on the oligonucleotides used for site-directed mutagenesis is shown in Supplementary Table S1. The integrity of all plasmids was verified by Sanger sequencing.

### Cell culture and transfection

HeLa and HCT-116 cells were obtained from RIKEN BioResource Research Center (Wako, Japan). Cells were grown in alpha-modified minimum essential medium (FUJIFILM Wako Pure Chemical) supplemented with 10% fetal bovine serum (Corning, USA), 100 µg/mL streptomycin, and 100 units/mL penicillin. Cells were cultured under standard conditions at 37 °C in a humidified incubator containing 5% CO_2_. Transfection of HeLa cells with the EGFP-TOP2B expression plasmid was performed using a FuGENE6 reagent (Promega, USA) according to the manufacturer’s instructions.

### Genome editing to generate a cell line harboring TOP2B H58Y

A cell line harboring the heterozygous H58Y substitution in TOP2B was generated by genome editing. A single-stranded oligodeoxynucleotide for the H58Y substitution together with single guide RNA and recombinant Cas9 was electrotransferred to HCT-116 cells. Cells harboring H58Y were screened and isolated as single colonies. An additional round of single-colony isolation was carried out to establish a cell line. The heterozygous H58Y substitution was confirmed by Sanger sequencing of both genomic DNA and cDNA samples prepared from the established cell line. Detailed procedures for genome editing, screening, and cloning were described in Supplementary Information.

### Fluorescence microscopy

Fluorescence microscopy was carried out as described previously^35^. In brief, an FV1200-IX83 laser scanning confocal microscope with an oil-immersed 60× objective (Olympus, Japan) was used for fluorescence microscopy. For live cell imaging, cells grown on a glass-bottomed dish were placed on a stage top incubator (Tokai Hit, Japan) that maintained a humidified atmosphere and 5% CO_2_ at 37 °C. Images were captured and analyzed using FLUOVIEW software (Version 4.1, Olympus).

### FRAP analysis

FRAP analysis was carried out as reported previously^35^. Briefly, HeLa cells in a glass bottom dish were transfected with EGFP-TOP2B plasmid DNA. At 48 h after transfection, live cell imaging was performed as described above. A spot in the nucleoplasm of a transfected cell was photobleached with a 473 nm laser at 35% output for 1 s. Before and after photobleaching, fluorescent images were acquired at regular intervals. Fluorescence intensities of photobleached and unbleached regions were quantified using FLUOVIEW software (Version 4.1, Olympus). All measurements in the photobleached areas were corrected for nonspecific bleaching during monitoring with reference to an unbleached area in the same cell. Fluorescence intensities before and immediately after photobleaching were set to 1 and 0, respectively. Fluorescence recovery was expressed as a ratio of fluorescence before and after photobleaching. For FRAP curve fitting by non-linear regression, we used the Stowers ImageJ plugins (https://research.stowers.org/imagejplugins/ImageJ_tutorial2.html) in the Fiji^58^ (ImageJ version 1.53t) according to the procedures described previously^59^. The normalized fluorescence recovery curves were fit with exponential recovery function using the ImageJ plugin ‘batch FRAP fit jru v1’ in the Stowers ImageJ plugins. A mobile fraction and t1/2 of fluorescence recovery were calculated for each curve.

### Western blot analysis

Cells were lysed in SDS-PAGE loading buffer containing 1% SDS. Cell lysates were cleared by brief sonication using a microsonicator (UR-20P, Tomy Seiko, Japan) and subsequent centrifugation. Cleared lysates were subjected to SDS-PAGE followed by electrotransfer to a PVDF membrane according to standard procedures. Proteins of interest were reacted with corresponding primary antibodies and detected by a chemiluminescence method using a secondary HRP-conjugated antibody and a Super Signal West Pico reagent (Thermo Fisher Scientific, USA). Chemiluminescence was detected using a ChemiDoc XRS Plus imaging system (Bio-Rad, USA) and quantified using ImageLab software (Version 2.0, Bio-Rad).

### Measurement of cell viability

Cells were plated in a 96-well plate and cultured at 37 °C overnight. After that, cells were treated with etoposide at concentrations indicated in the figure legend. After 2 h incubation at 37 °C, the etoposide-containing medium was removed, and cells were washed with a medium. Cell culture was continued in a fresh medium at 37 °C for 72 h. Cell viability was measured using a Cell Counting Kit-8 (Dojindo, Japan) and a microplate reader (MPR-A100, AsOne, Japan).

### Statistical analysis

Statistical analysis was carried out using Welch’s t-test. The number of experiments and p-values are indicated in the figure legends.

## Supplementary Information

Supplementary Information.

## Data Availability

The PDB codes used in Supplementary Fig. S1 were 1QZR (yeast TOP2 ATPase domain, https://www.ncbi.nlm.nih.gov/Structure/pdb/1QZR)^48^ and 7ZBG (human TOP2B ATPase domain, https://www.ncbi.nlm.nih.gov/Structure/pdb/7ZBG)^13^. The nucleotide sequence surrounding the codon 58 of wild-type TOP2B (Supplementary Fig. S2) was identical to the NCBI reference sequence NM_001068.2 (https://www.ncbi.nlm.nih.gov/nuccore/NM_001068.2/)^31^. The data that support the findings of this study are available from the corresponding authors upon reasonable request.
