# Supplementary material for: Disease-associated H58Y mutation affects the nuclear dynamics of human DNA topoisomerase IIβ
Source: Sci Rep. 2022 Nov 30;12:20627. doi: 10.1038/s41598-022-24883-2 (PMC9712534; doi:10.1038/s41598-022-24883-2)
Supplement: Supplementary file 1 — Supplementary Information. [file 41598_2022_24883_MOESM1_ESM.pdf]

Supplementary Information

Disease-associated H58Y mutation affects the nuclear dynamics of human DNA topoisomerase IIβ

Keiko Morotomi-Yano [1], Yukiko Hiromoto [2], Takumi Higaki [3,4] and Ken-ichi Yano [1,3] *

[1] Institute of Industrial Nanomaterials, Kumamoto University.

[2] Faculty of Science, Kumamoto University.

[3] Faculty of Advanced Science and Technology, Kumamoto University.

[4] International Research Organization for Advanced Science and Technology, Kumamoto University.

* Correspondence should be addressed to K. Y. (e-mail: yanoken@kumamoto-u.ac.jp)

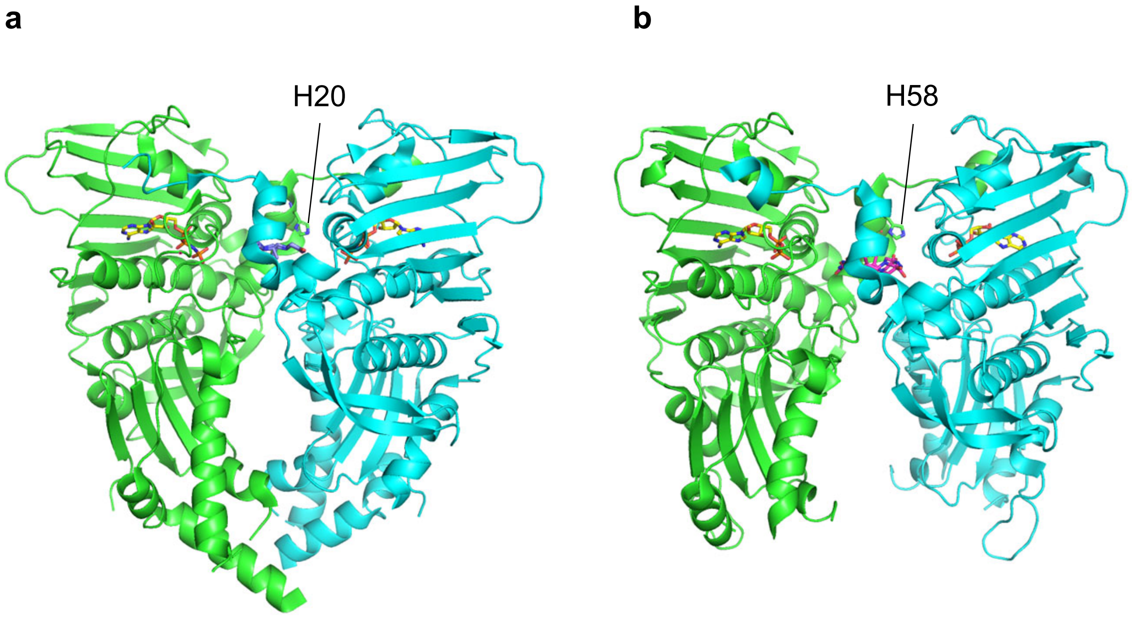

Supplementary Figure S1. ICRF-binding structures of yeast TOP2 and human TOP2B.

(a) Crystal structure of yeast TOP2 ATPase domain bound to ADP and ICRF-187 (PDB: 1QZR, Classen et al., 2003). Chains A and B of TOP2 ATPase homodimer are colored green and cyan, respectively. The image was generated with PyMOL (The PyMOL Molecular Graphics System, Version 2.5 Schrödinger, LLC.). (b) Crystal structure of human TOP2B ATPase domain bound to ADP and ICRF-193 (PDB: 7ZBG, Ling et al., 2022).

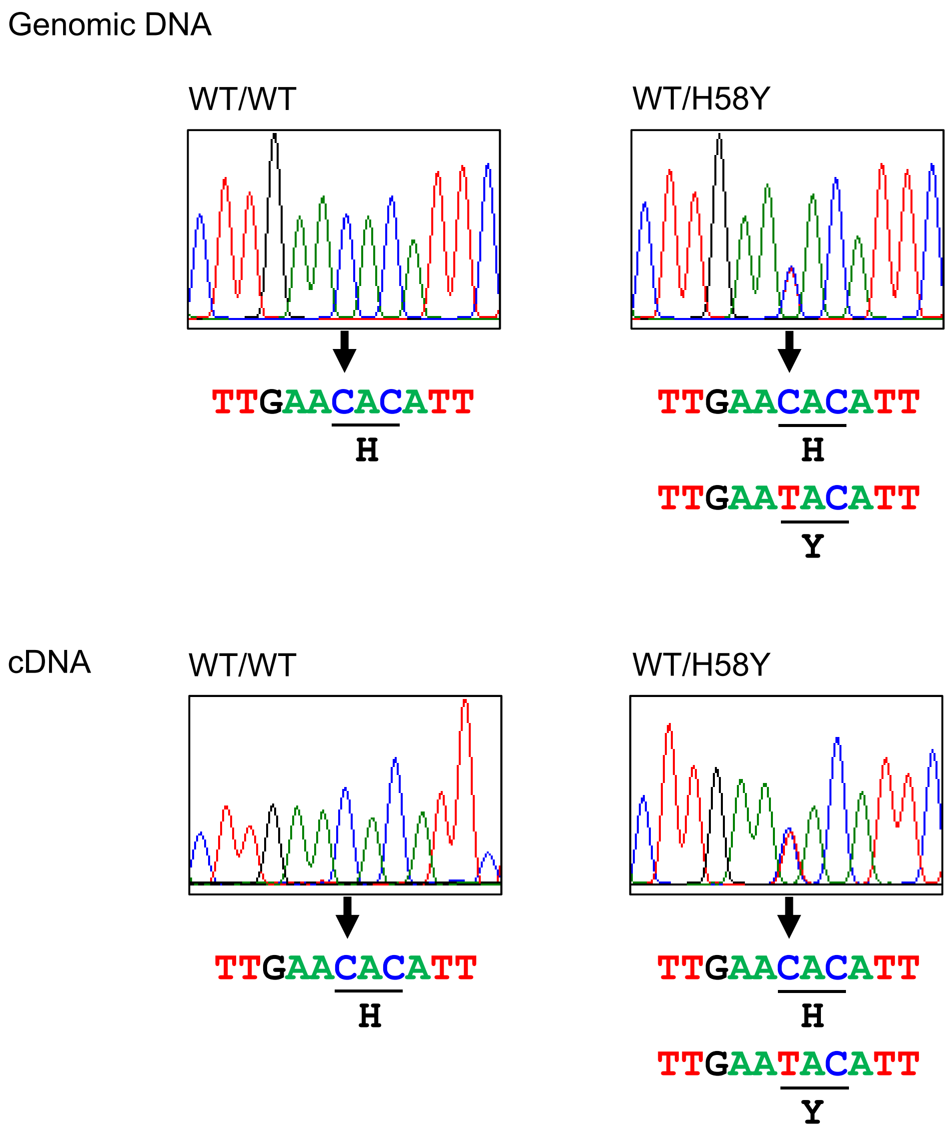
Genomic DNA
WT/WT
TTGAACACATT
H
WT/H58Y
TTGAACACATT
H
TTGAATACATT
Y
cDNA
WT/WT
TTGAACACATT
H
WT/H58Y
TTGAACACATT
H
TTGAATACATT
Y

Supplementary Figure S2. Validation of genome editing of the heterozygous H58Y substitution.

(Upper) Validation using genomic DNA. Genomic DNA was isolated from a cell line harboring a heterozygous H58Y substitution (WT/H58Y) and its parental HCT-116 cells (WT/WT). The region surrounding the codon 58 of TOP2B was amplified by PCR using the primers TOP2B-19228F and TOP2B-20154. The primer sequences are shown in Supplementary Table S1. PCR products were resolved by agarose gel electrophoresis, and the bands of interest were purified and subjected to Sanger sequencing. Representative electropherograms of Sanger sequencing are shown. Underlined letters indicate the codon 58. (Lower) Validation using cDNA. Total RNA was prepared from WT/WT and WT/H58Y cells and reverse-transcribed to synthesize cDNA. The region surrounding the codon 58 of TOP2B was amplified by PCR using the primers TOP2B-47F and TOP2B-840F. The primer sequences are shown in Supplementary Table S1. Sanger sequencing was performed as described above.

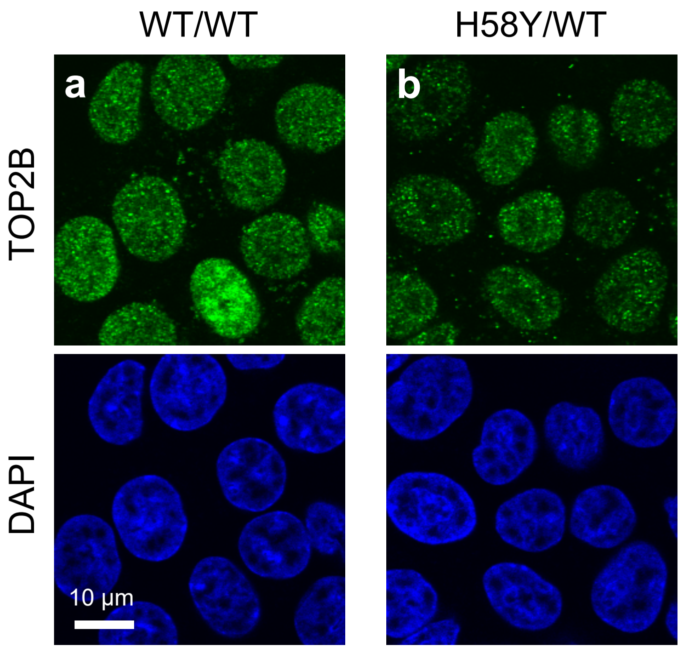

Supplementary Figure S3. Immunofluorescence staining of TOP2B in H58Y/WT and WT/WT cells.

WT/WT cells (a) and H58Y/WT (b) cells were used for immunostaining of TOP2B. Cells were washed with phosphate-buffered saline (PBS) and fixed with 4% paraformaldehyde on ice for 30 min. After washing with ice-cold PBS, cells were permeabilized with 0.1% Triton X-100 for 3 min. Cells were subsequently blocked with 1% bovine serum albumin solution for 15 min and reacted with an anti-TOP2B antibody (#611492, BD Biosciences, USA). After washing with PBS, cells were incubated with a secondary anbitody (anti-mouse IgG-Alexa Fluor488, A11029, Thermo Fisher Scientific, USA) and subsequently mounted in a Vectashield mounting medium containing DAPI (H-1200, Vector Laboratories, USA).

Experiment 1

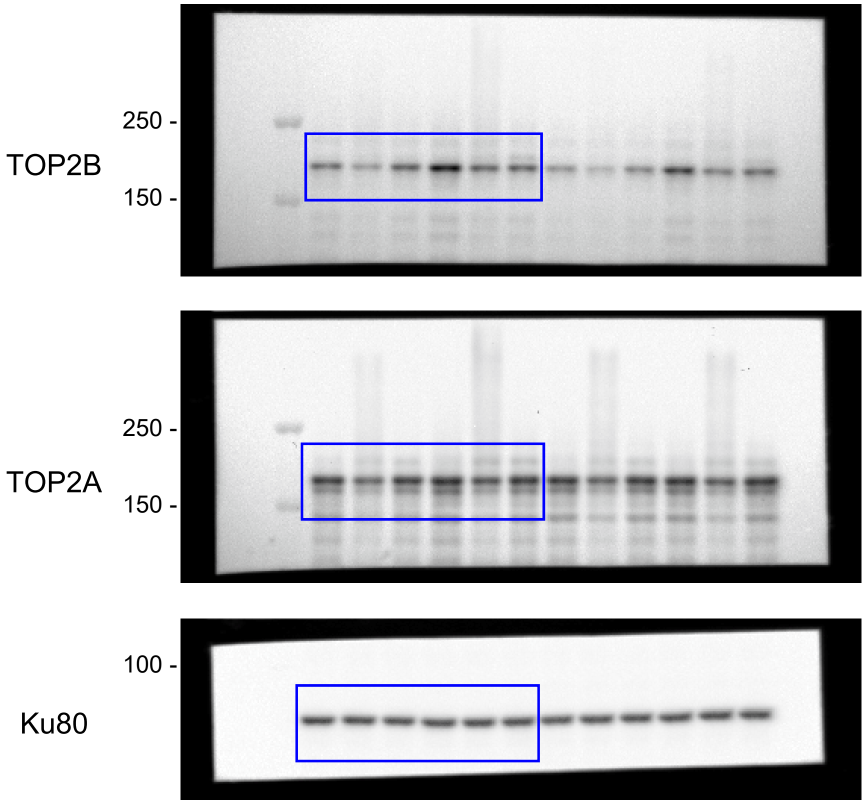

Supplementary Figure S4. Original uncropped images of the Western blots in Fig. 6.

The areas shown in Fig. 6a were indicated by blue boxes. Numbers on the left indicate molecular weights (kDa). Experiments were repeated three times, and the results of the second and third experiments are shown in the next page.

Experiment 2

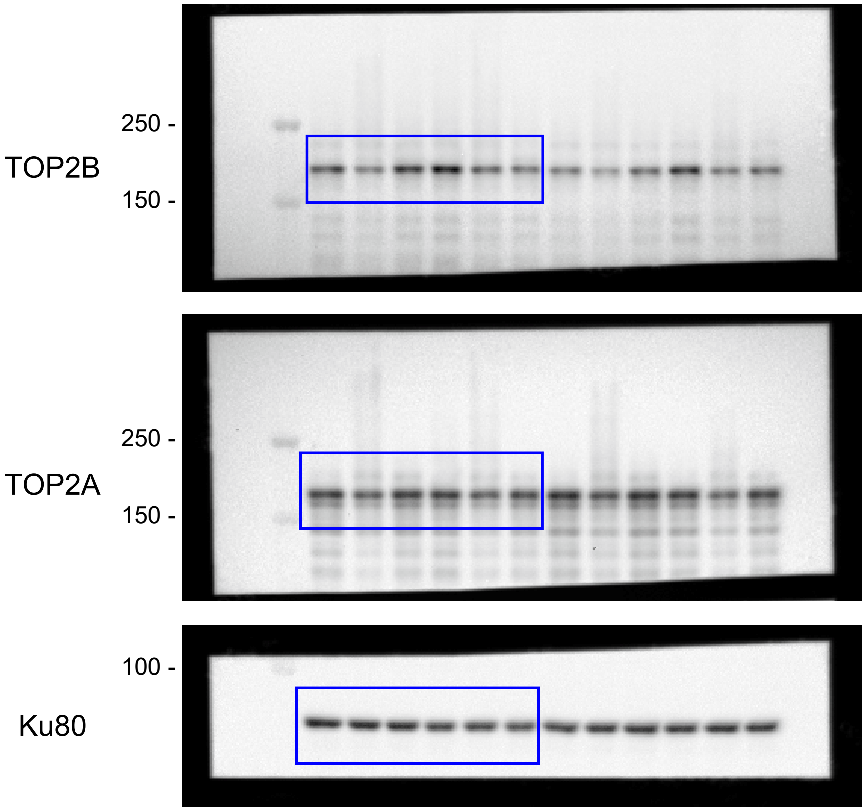

Experiment 3

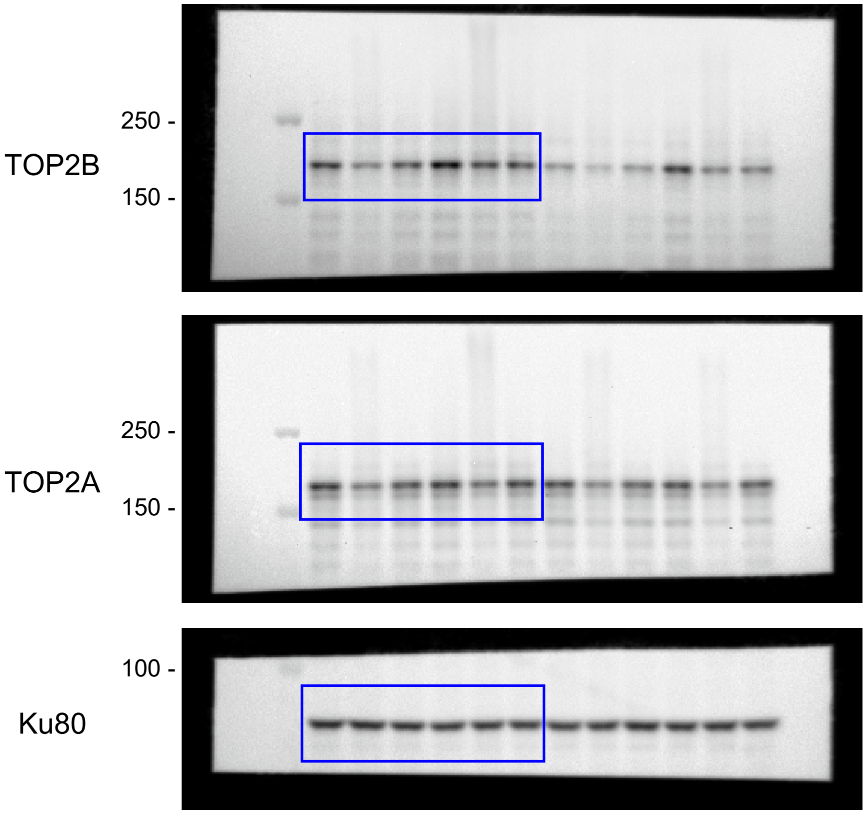

Supplementary Figure S4 (continued). Original uncropped images of the Western blots in Fig. 6. The results of the second and third experiments are shown. Average values of relative intensities of western blot bands were calculated from three experiments and used for Fig. 6b and 6c.

Supplementary Table S1. List of oligonucleotides used in this study.

Name	Sequence (5'-3')
TOP2B-H58Y-F	gaagacacaacttgaaTacattcttcttcgtcctg
TOP2B-H58Y-R	caggacgaagaagaatgtAttcaagttgtgtcttc
TOP2B-T65I-F	cctgataTatatattgggtcagtggagcc
TOP2B-T65I-R	aatatatAtatcaggacgaagaagaatgtg
TOP2B-Y66F-F	gatacatTtattgggtcagtggagccattg
TOP2B-Y66F-R	cccaataAatgtatcaggacgaagaagaatg
TOP2B-G180I-F	ttacaggtggtcgtaatATttatggtgcaaaactt
TOP2B-G180I-R	aagttttgcaccataaATattacgaccacctgtaa
TOP2B-L185F-F	tggttatggtgcaaaaTtttgtaatattttcagta
TOP2B-L185F-R	tactgaaaatattacaaaAttttgcaccataacca
TOP2B-sg-4	cctctaatacgactcactatagg<u>actgacccaatatatgtatc</u>gtttaagag ctatgc
TOP2B-ssODN	t*g*agagagtgtatcagaagaagacacaacttgaaTacattcttcttcgtc ctgatacGtatattgggtcagtggagccattgacgcaggtaattattg*c*t
TOP2B-19228F	tacccacgttttgggctcag
TOP2B-20154R	agttcgtatggcaccattttactc
TOP2B-47F	tcagggcctgtgagctggaggcac
TOP2B-840R	catgagggccacaatatccttgtc

Uppercase, mutated nucleotide; underline, TOP2B target sequence; *, phosphorothioate linkage.

Supplementary Methods

Generation of a cell line that harbors heterozygous TOP2B H58Y

Single guide RNA (sgRNA) was prepared and purified using a Guide-it Complete sgRNA Screening System (632636, Takara Bio, Japan). According to the manual of the Guide-it Complete sgRNA Screening System, a primer (TOP2B-sg-4) was designed, synthesized, and used for amplification of a DNA template for sgRNA. The amplified product was used for in vitro transcription, and the transcribed sgRNA was purified. A single-stranded oligodeoxynucleotide (TOP2B-ssODN) was designed and used as a donor DNA for the H58Y substitution. A SnaBI restriction site was included in TOP2B-ssODN for screening purposes.

For electrotransfer of the RNP complex, a mixture of 2 μg sgRNA and 9.9 μg Guide-it Recombinant Cas9 (632641, Takara Bio) were incubated at 37°C for 5 min to form an RNP complex. ssODN (6.6 μg, 200 pmol) was subsequently added to the RNP complex. HCT-116 cells were detached by treatment with Trypsin-EDTA and suspended in Opti-MEM (Thermo Fisher Scientific, USA). An aliquot of cell suspension (2×10^5 cells) was mixed with the RNP complex/ssODN and subjected to a single shot of 10 ms electric pulse at 140 V generated by a Gene Pulser Xcell Electroporation System (Bio-Rad, USA).

Individual cells were isolated as single colonies by limiting dilution. Cells derived from a single colony were lysed in solution containing 10 mM Tris 7.5, 150 mM NaCl, 5 mM EDTA, 0.5% NP40, 0.2 mg/ml Proteinase K. A pair of PCR primers (TOP2B-19228F and TOP2B-20154R) were designed for the amplification of the genome DNA corresponding to TOP2B H58. PCR was performed using the cell lysates. When the H58Y substitution was introduced into its corresponding genomic sequence, the PCR product should contain a SnaBI site, which was not present in the wild-type TOP2B sequence. Thus, PCR products were digested with SnaBI (R0130, New England Biolabs, USA) and resolved by agarose gel electrophoresis to examine the presence of the SnaBI site. PCR products that contained a SnaBI site were subsequently subjected to Sanger sequencing to confirm that genome editing yielded the H58Y substitution.

To further validate the heterozygous H58Y substitution, total RNA was prepared from cells and used for reverse-transcription to yield cDNA. PCR, SnaBI digestion, and Sanger sequencing were performed on cDNA as described above.

Sequence information on the oligonucleotides described above is shown in Supplementary Table S1.
